# Selected papers from the 12^th^ annual Bio-Ontologies meeting

**DOI:** 10.1186/2041-1480-1-S1-I1

**Published:** 2010-06-22

**Authors:** Larisa N Soldatova, Phillip Lord, Susanna-Assunta Sansone, Susie M Stephens, Nigam H Shah

**Affiliations:** 1 Aberystwyth University, Wales, UK; 2 Newcastle University, UK; 3 The European Bioinformatics Institute, Cambridge, UK; 4 Jonson & Johnson Pharmaceutical Research & Development LLC, PA, USA; 5 Stanford University, CA, USA

## Introduction and background

Biology, medicine, and biomedical computing have become critically dependent on the use of ontologies. Resources such as the Gene Ontology, the National Cancer Institute’s Thesaurus, the Foundational Model of Anatomy, SNOMED-CT, and the Ontology for Biomedical Investigation have become integral components of modern biomedical research and practice. Where once ontologies were perceived as arcane, over-complicated, and perhaps a bit over-hyped, they now serve as essential infrastructure for contemporary biology and medicine. In the past several years, the importance of ontologies in biomedicine has sky-rocketed. Ontologies are used to annotate experimental data, to aid information retrieval, to enable integration of heterogeneous data sets, to drive literature mining, and to build electronic knowledge bases. Recent research on biomedical ontologies and its application in life sciences focuses on data sharing standards, as well as semantic enrichment of existing scholarly content.

Bio-Ontologies has been a Special Interest Group (SIG) at ISMB for the last 12 years, providing a venue for sharing experiences and methods on the use of ontologies and their application to life sciences. Over the years, the Bio-Ontologies SIG has provided a forum for discussion on the latest and most innovative topics in this area. In 2009, the SIG received 27 paper submissions and 8 poster abstracts. 14 papers were selected for presentation at the meeting, out of which 7 papers have been selected for this special issue.

## Summary of selected papers

The seven papers selected for this special issue are extended versions of the original papers presented at the 2009 SIG. The papers span a wide range of topics including theoretical research, representation of biological and artifactual functions, an ontology for modeling biomedical experimental processes (OBI), an ontology for bibliographic referencing (CiTO), an ontology recommender web service, an approach for representation of biomedical statements (aTags), a knowledge Base for RNA structure and function, and a rule-based approach for semantic integration of heterogeneous data sources.

The paper titled “An Evolutionary Approach to Function” by Phillip Lord proposes new definitions of role and function different from those currently provided within the BFO (Basic Formal Ontology) [1]. Biological function is defined in terms of a realizable entity inhering in homologous structures in individuals of the same and closely related species. A formalization is provided for the definitions presented and applicability of the definition of function is elucidated with persuasive examples.

The paper “Building a Biomedical Ontology Recommender Web Service” by Clement Jonquet, Mark A. Musen and Nigam H. Shah introduces the ontology recommender web service at the National Center for Biomedical Ontology [2]. On the basis of user submitted textual corpus or keyword lists, the service recommends and ranks appropriate ontologies for use in annotation and curation tasks in that domain. The recommendation is based on three primary ranking criteria: coverage (how well the concepts in the ontology match the terms in the submitted text), connectivity (whether an ontology contains the terms most frequently referred to by other ontologies), and size (the number of concepts in an ontology). Such a service is sorely needed given that the number of available biomedical ontologies continues to grow. The service is available at: http://www.bioontology.org/ontology-recommender.

The paper “Modeling biomedical experimental processes with OBI” presented by the OBI Consortium – which combines 19 biomedical communities from around the globe – reports the progress on the development of the Ontology for Biomedical Investigations (OBI) [3]. OBI addresses the need for the integrated standards for the reporting of biological and clinical investigations. The paper describes the key elements of OBI and explains how to use logically defined OBI descriptors to model experimental process. Three examples from biomedical domains are discussed: a neuroscience experiment, a vaccine study, and an automated functional genomics investigation. These use cases help to specify competency questions, to validate OBI's design choices, and to identify areas of OBI that need further development. OBI is available at: http://obi-ontology.org.

In the paper “CiTO, the Citation Typing Ontology, and its use for annotation of reference lists and visualization of citation networks”, David Shotton reports the results of the work on the representation of different types of scientific citations using the Citation Typing Ontology (CiTO) [4]. CiTO enables creation of scientific citations in machine readable form on the Semantic Web, characterizing the cited work and defining relations between citing and cited papers. CiTO extends the Functional Requirements for Bibliographic Records (FRBR) classification for works, expressions, and manifestations. The author integrates CiTO with several existing formalisms, such as the Dublin Core metadata, FRBR, and the Semantic Web Applications in Neuroscience (SWAN) ontology. If used globally, CiTO holds the potential to have a significant impact on the publication of scientific literature. CiTO is available at: http://purl.org/net/cito/.

Allyson Lister, Phillip Lord, Matthew Pocock and Anil Wipat in their paper “Annotation of SBML Models Through Rule-Based Semantic Integration” describe a novel method for the rule-based semantic integration of heterogeneous data sources with the use of a core ontology [5]. The method is explained and demonstrated on examples from models represented in the Systems Biology Markup Language. The proposed method can be applied to various domains.

The paper “RKB: A Semantic Web Knowledge Base for RNA” by Jose Cruz-Toledo, Michel Dumontier, Marc Parisien and Francois Major describes the creation of an RNA knowledge base RKB for structure-based knowledge using Semantic Web technologies [6]. RKB extends a number of ontologies (such as the Information Artefacts Ontology (IAO) and ChEBI) and contains a basic terminology for ribonucleic acid composition along with context/model-specific structural features such as sugar conformations, base pairings and base stackings. The authors demonstrate queries to the RKB using description logic reasoning; thus enabling question answering over knowledge about RNA using semantic web technologies. RKB is available at http://semanticscience.org/projects/rkb.

The paper “Simple, Ontology-Based Representation of Biomedical Statements through Fine-Granular Entity Tagging and New Web Standards” by Matthias Samwald and Holger Stenzhorn represents a step forward to making semantic web technology more widely accessible to users [7]. The paper describes aTags (‘associative tags’) which are short snippets of HTML+RDFa with embedded RDF/OWL based on the Semantically Interlinked Online Communities (SIOC) vocabulary and domain ontologies and taxonomies. To foster adoption of the aTag system, the authors also present tools and services to allow a user to tag items as well as seed the system with a number of datasets marked up with aTags. The system is available at http://hcls.deri.org/atag.

## Competing interests

The authors declare that they have no competing interests.
